# Racial and Ethnic Disparities in EMS Use of Restraints and Sedation for Patients With Behavioral Health Emergencies

**DOI:** 10.1001/jamanetworkopen.2025.1281

**Published:** 2025-03-20

**Authors:** Diana M. Bongiorno, Gregory A. Peters, Margaret E. Samuels-Kalow, Scott A. Goldberg, Remle P. Crowe, Anjali Misra, Rebecca E. Cash

**Affiliations:** 1 Harvard Medical School, Boston, Massachusetts; 2 Department of Emergency Medicine, Massachusetts General Hospital, Boston; 3 Department of Emergency Medicine, Brigham and Women’s Hospital, Boston, Massachusetts; 4 ESO Solutions, Austin, Texas

## Abstract

**Importance:**

Emergency medical services (EMS) clinicians commonly care for patients with behavioral health emergencies (BHEs), including acute agitation. There are known racial and ethnic disparities in the use of physical restraint and chemical sedation for BHEs in emergency department settings, but less is known about disparities in prehospital use of restraint or sedation.

**Objective:**

To investigate the association of patient race and ethnicity with the use of prehospital physical restraint and chemical sedation during EMS encounters for BHEs.

**Design, Setting, and Participants:**

This nationwide retrospective cohort study used data from EMS agencies across the US that participated in the 2021 ESO Data Collaborative research dataset. Emergency medical services encounters among patients aged 16 to 90 years with a primary or secondary impression, sign or symptom, or protocol use associated with a BHE from January 1 to December 31, 2021, were included. Statistical analysis was conducted from July 2023 to March 2024.

**Exposures:**

Patient race and ethnicity, which was categorized as Hispanic, non-Hispanic Black, non-Hispanic White, non-Hispanic other (American Indian or Alaska Native, Asian, Hawaiian Native or Other Pacific Islander, other, or multiracial), and unknown.

**Main Outcomes and Measures:**

The primary outcome was administration of any physical restraint and/or chemical sedation (defined as any antipsychotic medication, benzodiazepine, or ketamine).

**Results:**

A total of 661 307 encounters (median age, 41 years [IQR, 30-56 years]; 56.9% male) were included. Race and ethnicity were documented as 9.9% Hispanic, 20.2% non-Hispanic Black, 59.5% non-Hispanic White, 1.9% non-Hispanic other, and 8.6% unknown race and ethnicity. Restraint and/or sedation was used in 46 042 (7.0%) of encounters, and use differed across racial and ethnic groups (Hispanic, 10.6%; non-Hispanic Black, 7.9%; non-Hispanic White, 6.1%; non-Hispanic other, 10.9%; unknown race and ethnicity, 5.9%; *P* < .001). In mixed-effects logistic regression models accounting for clustering by EMS agency and adjusted for age, gender, urbanicity, and community diversity, patients who were non-Hispanic Black had significantly greater odds of being restrained or sedated across all categories compared with non-Hispanic White patients (eg, any restraint and/or sedation: adjusted odds ratio [AOR], 1.17 [95% CI, 1.14-1.21]; physical restraint: AOR, 1.22 [95% CI, 1.18-1.26]). There was no significant difference in adjusted odds of any restraint and/or sedation use for the remaining racial and ethnic groups compared to non-Hispanic White patients. Clustering was associated with agency-level variation in restraint or sedation use (intraclass correlation coefficient, 0.16 [95% CI, 0.14-0.17]).

**Conclusions and Relevance:**

This nationwide retrospective cohort study of EMS encounters for patients with BHEs found differences in the use of prehospital restraint and/or sedation by patient race and ethnicity and an agency-level association with variation in restraint and/or sedation use. These data may inform improvements to protocols and training aimed at equitable care for BHEs.

## Key Points

**Question:**

Are there racial and ethnic disparities in the use of physical restraint and/or chemical sedation in the prehospital setting for patients with behavioral health emergencies (BHEs)?

**Finding:**

In this cohort study of 661 307 emergency medical services (EMS) BHE encounters, non-Hispanic Black patients had significantly greater odds of being restrained and/or sedated compared with non-Hispanic White patients; there was no significant difference in odds of any restraint and/or sedation use for other racial and ethnic groups compared with non-Hispanic White patients. Clustering by EMS agency was associated with variation in restraint and/or sedation use.

**Meaning:**

This study suggests that racial and ethnic differences in restraint and sedation use may represent care disparities and opportunities for improving equity, such as by improving EMS protocols and training for BHEs.

## Teaser

This nationwide cohort study investigates the association of patient race and ethnicity with the use of prehospital physical restraint and chemical sedation during emergency medical services (EMS) encounters for patients with behavioral health emergencies.

## Introduction

An estimated 7% of emergency medical services (EMS) activations are for patients with behavioral health emergencies (BHEs), including agitation.^1^ Caring for patients with acute agitation is particularly challenging in the prehospital setting, as EMS clinicians must consider safety on scene and within the enclosed space of an ambulance, often with limited resources.^2^ When safety of the clinicians, bystanders, and patient are at risk, the use of physical restraints and/or chemical sedation may be necessary.^2,3^ Although restraints and/or sedation are needed in certain situations, there are notable risks associated with these interventions, including respiratory depression, hypoxia, physical trauma, and, rarely, cardiac arrest.^2,4,5^ Restraint use can also contribute to patient psychological distress and distrust in the health care system.^6^ Given these risks, the National Association of EMS Physicians published a position statement in 2021 calling for EMS agencies to have protocols to guide prehospital management of BHEs, including use of physical restraint and chemical sedation.^2^

There is evidence of racial and ethnic disparities in the use of restraint and sedation within emergency department (ED) settings, but even less is known about prehospital disparities in the use of restraint or sedation. Several studies of ED patients with BHEs demonstrated that patients who are Black or Hispanic had significantly greater adjusted odds of physical restraint relative to White patients.^7,8,9^ However, the analyses could not differentiate between patients with physical restraints applied by EMS and those with physical restraints initiated in the ED. Fewer studies have investigated disparities in chemical sedation use. Black patients presenting to the ED with BHEs may have higher odds of chemical sedation, relative to non-Hispanic White patients, and there is conflicting evidence regarding odds of chemical sedation use among Hispanic patients.^10,11^

Given the adverse effects associated with the use of restraint and sedation, as well as the possibility that prehospital restraint and/or sedation could bias or otherwise affect subsequent ED care, it is important to understand whether racial and ethnic disparities in the use of physical restraint and chemical sedation exist in the prehospital setting. This study investigated the association of patient race and ethnicity with prehospital use of physical restraint and chemical sedation during EMS encounters for patients with BHEs.

## Methods

### Study Design and Data Source

We conducted a retrospective cohort study of EMS encounters for BHEs using the ESO Data Collaborative research dataset from January 1 to December 31, 2021. ESO, a large vendor of prehospital electronic health record software, offers this dataset in a public-release format using patient records from approximately 2000 agencies across all geographic census regions in the US.^12^ Organizations using ESO electronic health record software may voluntarily agree to have their deidentified health records included in the research dataset. Data were collected in compliance with the National Emergency Medical Services Information System data standard, and patient consent was waived as the data are deidentified. This study was reviewed by the Mass General Brigham institutional review board and was deemed exempt due to the deidentified nature of the dataset. We followed the Strengthening the Reporting of Observational Studies in Epidemiology (STROBE) reporting guideline for reporting this cohort study.

### Study Population

We included EMS encounters for patients aged 16 to 90 years with BHEs who were transported by ground to a health care facility after a 911 call. We excluded encounters that were not 911 activations to the scene (eg, interfacility transfers), not treated and transported by the same unit, those transported by air, and those with indication of a seizure. Given the data are deidentified, the unit of analysis is the encounter rather than the patient. We excluded air transports, given that the threshold for restraint or sedation may differ during flight. Encounters with indication of a seizure, based on primary or secondary impression, sign or symptom, or protocol, were excluded because benzodiazepines or ketamine may have been used for seizure treatment rather than sedation.

Emergency medical services encounters for BHEs were identified by EMS clinicians’ primary or secondary impressions (eg, behavioral or psychiatric episode, suicidal ideation), sign or symptom (eg, combative or violent behavior, hallucinations), or protocol use (eg, behavioral or overdose/toxic ingestion) (eTable 1 in Supplement 1). We included encounters for patients with substance use disorders, as similar studies of ED restraint use have demonstrated inequities in care for these patients.^8,13^

For secondary analyses that included the outcome of chemical sedation, we further limited the sample to advanced life support (ALS) units, typically staffed by paramedics.^4^ Basic life support (BLS) units, typically staffed by emergency medical technicians, generally may use physical restraints, but not chemical sedation, within their scope of practice. We defined ALS units as those staffed by 1 or more paramedics. Units designated as advanced emergency medical technicians (often considered ALS but unable to administer chemical sedation) or with missing data on unit level of care were excluded from analyses related to chemical sedation.

### Measures

The primary outcome was use of any physical restraint and/or chemical sedation. Physical restraint was defined as documentation of physical restraint application or restraint use as a barrier to care. Chemical sedation was defined as administration of ketamine, benzodiazepines (midazolam, lorazepam, diazepam), or antipsychotic agents (haloperidol, droperidol, olanzapine, ziprasidone) by any route (eg, intramuscular, intravenous, oral).^2,4,10,14,15,16^ Secondary outcomes of interest were (1) any physical restraint use (ie, with or without chemical sedation during that encounter), (2) any chemical sedation use, and (3) use of both physical restraint and chemical sedation in the same encounter.

The primary independent variable was patient race and ethnicity. Recognizing that race is a social construct, a composite variable of race and ethnicity was used as our variable of interest as a measure of implicit bias and/or structural racism. Race and ethnicity are typically recorded in EMS data based on EMS clinician impression rather than patient self-identification. Despite potential inaccuracies with regard to a patient’s true racial and ethnic identity, the EMS clinician’s impression of the patient’s race and ethnicity captures their perception of the patient and potential bias. Race and ethnicity are documented separately by the EMS clinician. We defined the patient’s race and ethnicity as one composite variable of Hispanic, non-Hispanic Black, non-Hispanic White, non-Hispanic other, and unknown. These categories were informed by prior studies investigating ED restraint use.^7,10^ Patients recorded as “Hispanic/Latino” in the ethnicity variable, race variable, or both were categorized as Hispanic. Races of American Indian or Alaska Native, Asian, Hawaiian Native or Other Pacific Islander, other, or multiracial were collapsed into the non-Hispanic other subgroup for sample size purposes due to the low prevalence of each. Unknown race and ethnicity included patients who were missing information for both race and ethnicity. Missing race and ethnicity data are likely informative and potentially related to implicit bias. Therefore, we included those with missing data, in alignment with prior research on restraint use.^7^

Other variables of interest included additional patient demographic characteristics, neighborhood-level factors, and EMS agency. Patient age was categorized as 16 to 24, 25 to 34, 35 to 44, 45 to 54, 55 to 64, and 65 to 90 years.^7^ Patient gender was defined in the ESO dataset as male, female, or unknown or missing. Encounter urbanicity was defined as urban vs suburban or rural. To further account for potential bias and structural factors associated with the neighborhood to which EMS responded, we incorporated the Centers for Disease Control and Prevention Social Vulnerability Index (SVI) theme for racial and ethnic minority status for the incident location. The SVI racial and ethnic minority status theme is derived from the percentile ranking of the proportion of racial and ethnic minority populations in that US Census tract relative to all counties in the US.^17^ Higher SVI corresponds to greater social vulnerability. We categorized SVI into quartiles for each encounter (<25th percentile, 25-49th percentile, 50-74th percentile, and ≥75th percentile). Finally, we used a masked agency identifier to identify encounters associated with the same EMS agency.

### Statistical Analysis

Statistical analysis was conducted from July 2023 to March 2024. We first described demographic, neighborhood-level, and agency characteristics as well as each outcome across race and ethnicity groups. We used Pearson χ^2^ tests to compare each restraint and sedation outcome by racial and ethnic group. We fit separate mixed-effects logistic regression models to estimate the odds of each outcome by racial and ethnic group. Given the potential role of the EMS agency and agency-level protocols and organizational culture, mixed-effects logistic regression was selected to enable us to evaluate the effect of EMS agency for each model through calculation of intraclass correlation coefficients (ICCs). We a priori identified potential confounding variables, including patient age, gender, urbanicity, and the SVI racial and ethnic minority status theme. Variance inflation factors were calculated for each multivariable model to confirm that multicollinearity was not present.

We performed a sensitivity analysis restricting the definition of chemical sedation to encounters with administration of at least 1 medication of interest (ie, ketamine, benzodiazepines, and/or antipsychotic agents) by intramuscular route, as intramuscular medications may be used more often for patients with the most acute agitation and certain prior studies have restricted the definition of sedation to intramuscular medications.^4,11^ Missing data were handled using available case analysis. We performed all statistical analyses using Stata SE, version 16.1 (StataCorp LLC) and R, version 3.6.2 (R Foundation for Statistical Computing). All *P* values were from 2-sided tests, and results were deemed statistically significant at *P* < .05.

## Results

### Characteristics of Study Participants

**Figure 1.**
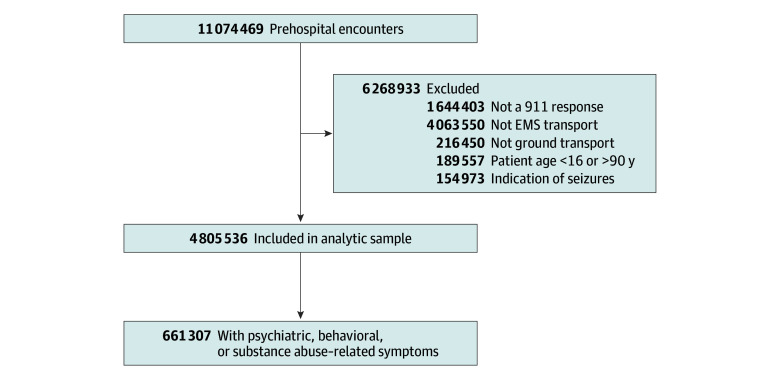
Flow Diagram for Emergency Medical Services (EMS) Encounters Included in Study Population

In total, 661 307 EMS encounters related to psychiatric, substance-related, or other behavioral concerns were included in the final analytic sample (Figure 1), which included patients documented as Hispanic (65 214 [9.9%]), non-Hispanic Black (133 305 [20.2%]), non-Hispanic White (393 305 [59.5%]), and non-Hispanic other (12 883 [1.9%]) (Table 1). Race and ethnicity were unknown in 56 600 encounters (8.6%). The median age of patients was 41 years (IQR, 30-56 years); of the 658 515 patients with gender data, 283 493 (43.1%) were female, and 375 022 (56.9%) were male. Most encounters were in urban settings (562 778 of 660 305 [85.2%]) and managed by ALS units (569 662 of 660 344 [86.3%]).

**Table 1.** Characteristics of Patients and EMS Encounters

Characteristic	All	Hispanic	Non-Hispanic Black	Non-Hispanic White	Non-Hispanic Other	Unknown
Total, No. (%)	661 307 (100)	65 214 (9.9)	133 305 (20.2)	393 305 (59.5)	12 883 (1.9)	56 600 (8.6)
Age, median (IQR), y	41 (30-56)	35 (26-48)	38 (28-53)	43 (31-58)	36 (26-50)	40 (29-55)
Age group, No. (%), y						
16-24	94 746 (14.3)	13 961 (21.4)	21 925 (16.4)	47 100 (12.0)	2711 (21.0)	9049 (16.0)
25-34	148 274 (22.4)	17 291 (26.5)	34 078 (25.6)	80 724 (20.5)	3174 (24.6)	13 007 (23.0)
35-44	133 975 (20.3)	13 786 (21.1)	26 832 (20.1)	79 328 (20.2)	2664 (20.7)	11 365 (20.1)
45-54	103 854 (15.7)	9816 (15.1)	20 132 (15.1)	63 260 (16.1)	1950 (15.1)	8696 (15.4)
55-64	98 025 (14.8)	6626 (10.2)	19 154 (14.4)	63 249 (16.1)	1282 (10.0)	7714 (13.6)
65-90	82 433 (12.5)	3734 (5.7)	11 184 (8.4)	59 644 (15.2)	1102 (8.6)	6769 (12.0)
Gender, No. (%)						
Female	283 493 (43.1)	23 968 (36.8)	54 503 (40.9)	175 289 (44.6)	5657 (44.0)	24 076 (44.4)
Male	375 022 (56.9)	41 199 (63.2)	78 725 (59.1)	217 730 (55.4)	7214 (56.0)	30 154 (55.6)
Missing^a^	2792	47	77	286	12	2370
Urbanicity, No. (%)						
Suburban or rural	97 527 (14.8)	5772 (8.9)	10 438 (7.8)	73 851 (18.8)	1376 (10.7)	6090 (10.8)
Urban	562 778 (85.2)	59 242 (91.1)	122 722 (92.2)	318 906 (81.2)	11 487 (89.3)	50 421 (89.2)
Missing^a^	1002	200	145	548	20	89
SVI racial and ethnic minority status theme, No. (%)						
0-24th Percentile	130 808 (19.8)	4466 (6.9)	9641 (7.2)	104 631 (26.7)	1362 (10.6)	10 708 (19.0)
25th-49th Percentile	180 378 (27.3)	10 119 (15.6)	31 959 (24.0)	118 868 (30.3)	3030 (23.6)	16 402 (29.0)
50th-74th Percentile	193 054 (29.3)	16 889 (26.0)	47 949 (36.0)	107 656 (27.4)	3955 (30.8)	16 605 (29.4)
75th-99th Percentile	155 732 (23.6)	33 486 (51.5)	43 592 (32.7)	61 363 (15.6)	4514 (35.1)	12 777 (22.6)
Missing^a^	1335	254	164	787	22	108
EMS level of care, No. (%)						
ALS	569 662 (86.3)	56 141 (86.2)	112 914 (84.8)	349 340 (88.9)	9924 (77.5)	41 343 (73.1)
BLS	90 682 (13.7)	9001 (13.8)	20 162 (15.2)	43 415 (11.1)	2876 (22.5)	15 228 (26.9)
Missing^a^	963	72	229	550	83	29

Abbreviations: ALS, advanced life support; BLS, basic life support; EMS, emergency medical services; SVI, Social Vulnerability Index.

^a^ Missing values are not included in the denominators for percentages calculated in each category.

### Main Results

Overall, physical restraint and/or chemical sedation was used in 46 042 encounters (7.0%). Among the 661 307 included encounters, use of any physical restraint (29 874 [4.5%]) was more common than any chemical sedation use (25 614 [3.9%]); both restraint and sedation were used in 9446 encounters (1.4%). Among ALS unit encounters with chemical sedation administration, most involved the use of benzodiazepines (71.3% [18 140 of 25 434]), compared with 27.0% for antipsychotic agents (6878 of 25 434) and 17.7% for ketamine (4505 of 25 434). There were differences noted in the proportion of any restraint and/or sedation use across racial and ethnic subgroups (Hispanic, 10.6% [6908 of 65 214]; non-Hispanic Black, 7.9% [10 483 of 133 305]; non-Hispanic White, 6.1% [23 926 of 393 305]; non-Hispanic other [1407 of 12 883], 10.9%; unknown race and ethnicity, 5.9% [3318 of 56 600]; *P* < .001) (Table 2).

**Table 2.** Proportion of Encounters With Physical Restraint, Chemical Sedation, or Both, Stratified by Race and Ethnicity

Encounter	No. (%)					*P* value^a^
	Hispanic (n = 65 214)	Non-Hispanic Black (n = 133 305)	Non-Hispanic White (n = 393 305)	Non-Hispanic other (n = 12 883)	Unknown (n = 56 600)	
Any restraint or sedation	6908 (10.6)	10 483 (7.9)	23 926 (6.1)	1407 (10.9)	3318 (5.9)	<.001
Any physical restraint	4988 (7.7)	7342 (5.5)	14 213 (3.6)	1096 (8.5)	2235 (4.0)	<.001
Any chemical sedation	3489 (5.4)	5510 (4.1)	14 578 (3.7)	501 (3.9)	1536 (2.7)	<.001
Both restraint and sedation	1569 (2.4)	2369 (1.8)	4865 (1.2)	190 (1.5)	453 (0.8)	<.001

^a^ Calculated using Pearson χ^2^ tests.

**Figure 2.**
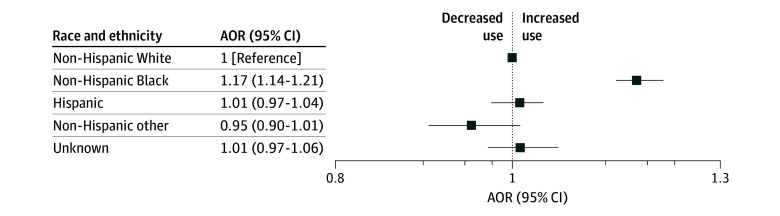
Adjusted Odds of Any Restraint and/or Sedation Use by Patient Race and Ethnicity Among Emergency Medical Services Encounters for Behavioral Health Emergencies Models accounted for clustering by emergency medical services agency and adjusted for age, gender, urbanicity, and community diversity (Social Vulnerability Index racial and ethnic minority status theme). AOR indicates adjusted odds ratio.

In unadjusted models accounting for clustering by agency, there were significantly greater odds of the primary outcome of any restraint and/or sedation use in encounters among non-Hispanic Black patients (odds ratio [OR], 1.24 [95% CI, 1.21-1.28]), Hispanic patients (OR, 1.11 [95% CI, 1.07-1.14]), and those with unknown race and ethnicity (OR, 1.05 [95% CI, 1.01-1.11]) compared with non-Hispanic White patients (eFigure in Supplement 1). In the fully adjusted model, patients who were non-Hispanic Black had significantly greater odds of any restraint and/or sedation (adjusted OR [AOR], 1.17 [95% CI, 1.14-1.21]), while there was no significant difference in adjusted odds of any restraint and/or sedation for patients of any other racial and ethnic groups, compared with non-Hispanic White patients (Figure 2). Clustering by agency was associated with agency-level variation in any restraint and/or sedation use (ICC, 0.16 [95% CI, 0.14-0.17]).

**Figure 3.**
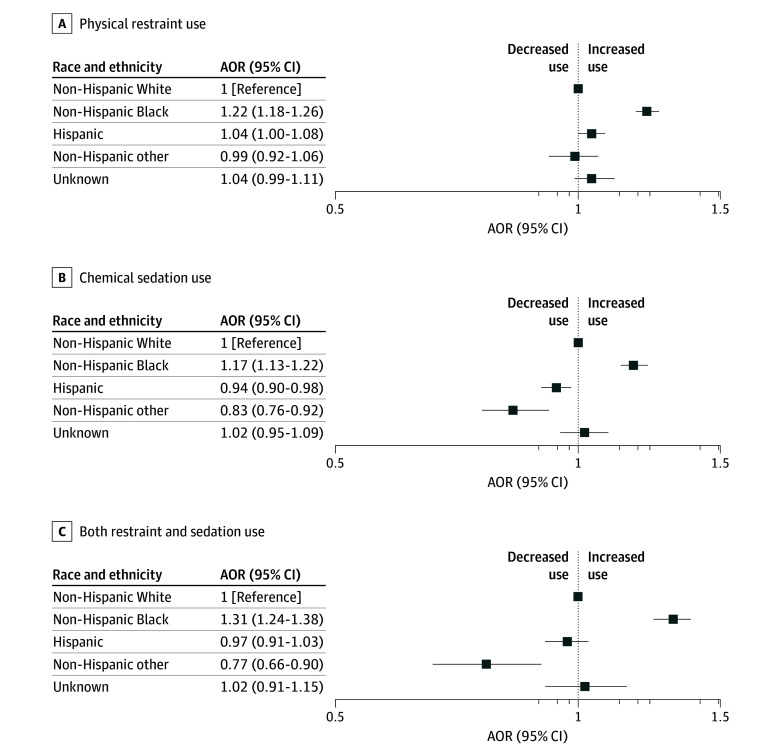
Adjusted Odds of Secondary Outcomes by Patient Race and Ethnicity Among Emergency Medical Services Encounters for Behavioral Health Emergencies Models accounted for clustering by emergency medical services agency and adjusted for age, gender, urbanicity, and community diversity (Social Vulnerability Index racial and ethnic minority status theme). AOR indicates adjusted odds ratio.

Patients who were non-Hispanic Black also had significantly greater adjusted odds of restraint and/or sedation use across all secondary outcomes (eg, physical restraint: AOR, 1.22 [95% CI, 1.18-1.26]; both restraint and sedation: AOR, 1.31 [95% CI, 1.24-1.38]) (Figure 3). Hispanic patients had significantly greater odds of being physically restrained (AOR, 1.04 [95% CI, 1.003-1.083]) compared with non-Hispanic White patients, but the same association was not seen in analyses of chemical sedation or both restraint and sedation. Patients in the non-Hispanic other group had significantly lower adjusted odds of receiving chemical sedation or both restraint and sedation compared with non-Hispanic White patients. In each of these models, clustering by agency was also associated with variation in restraint and sedation use (eg, chemical sedation: ICC, 0.25 [95% CI, 0.23-0.27]).

In sensitivity analyses restricting chemical sedation to the intramuscular route, overall restraint and/or sedation was used in 38 160 encounters (5.8%), chemical sedation was used in 14 562 encounters (2.2%), and both restraint and sedation were used in 6276 encounters (0.9%). The fully adjusted models yielded overall similar results as the primary analysis (eg, any restraint and/or sedation for non-Hispanic Black compared with non-Hispanic White patients: AOR, 1.24 [95% CI, 1.20-1.27]) (eTable 2 in Supplement 1).

## Discussion

In this nationwide study of EMS encounters, we found evidence of racial and ethnic disparities in the prehospital use of physical restraint and/or chemical sedation for patients with BHEs. Non-Hispanic Black patients had significantly greater odds of being restrained and/or sedated in a prehospital setting compared with non-Hispanic White patients. These findings are consistent with studies demonstrating that Black patients are more likely to receive physical restraints^7,8,9^ or chemical sedation^10,11^ in ED settings. We found no significant difference in adjusted odds of any restraint and/or sedation use for Hispanic compared with non-Hispanic White patients, although Hispanic patients did have significantly greater odds of being physically restrained. Similarly, among ED patients with BHEs, there is some evidence that Hispanic patients have greater odds of being physically restrained,^7,8^ although analyses of sedation administration have had conflicting results.^10,11^ It is possible that variation in the literature regarding restraint and sedation disparities among Hispanic patients could be due to differences in how Hispanic individuals were identified (ie, self-reported identity vs clinician impression, as in our study).

The disparities observed in our study are likely due to a combination of factors. Clustering by agency was associated with variation in restraint and/or sedation use, suggesting that agency-level practices (eg, agency protocols, organizational culture, and/or training) may play a role.^2^ Although EMS education programs follow the National EMS Education Standards, which include education on BHEs,^18^ there is no national standard for de-escalation training for EMS clinicians. One study using simulated cases to assess EMS clinicians’ response to threats of violence found that only approximately half had adequate verbal de-escalation attempts, while those who completed crisis intervention training had over twice the odds of attempting verbal de-escalation.^19^ Disparities may also be associated with other agency-level structural factors. One national study of ED patients found that Black patients had significantly greater adjusted odds of receiving chemical sedation, but this association was no longer significant after accounting for the hospital’s racial composition, as treatment in a hospital with a greater proportion of Black patients was associated with greater odds of receiving sedation.^10^ Similarly, EMS agencies that treat a higher proportion of Black patients could have greater odds of restraint or sedation use. Patients from certain racial and ethnic groups with BHEs may also present with more severe illness and therefore require restraint or sedation more often; for example, Black patients are less likely to have access to outpatient mental health care and are less commonly treated with psychotropic medications compared with White patients.^20,21^ In addition, disparities may be partly due to implicit bias, which can influence patient interactions and treatment.^22,23^ One study of ED patients found significant differences in rates of physical restraint administration across individual attending physicians,^24^ potentially due to practice variations and/or implicit bias that could similarly exist in the prehospital setting.

Prehospital restraint and sedation have the potential to limit patients’ initial ED evaluation or otherwise affect their ED care. Our results raise concern that prehospital disparities in restraint and sedation may contribute to disparities in ED settings. Most studies of ED patients with BHEs have not differentiated between restraints that are initiated in the ED or first applied by EMS. If an ED patient arrives with restraints already in use, it may be more likely that these restraints are continued when verbal de-escalation might otherwise have been attempted. Literature supports that prehospital factors impact ED restraint use. One multicenter study found that ED patients who arrived accompanied by police had over 5 times the odds of receiving physical restraints in the ED, and approximately 10% of the disparity in ED restraint use for non-Hispanic Black patients was mediated by police involvement in transport.^25^ Although confounding by indication may be at play (ie, police are more likely to be involved in higher-risk encounters), it is possible that law enforcement involvement in prehospital care may have similarly played a role in our findings. This is an important area for further investigation.

To address the prehospital disparities demonstrated in our study, future work should include investigation of EMS agency protocols for restraint or sedation use. Particularly given the EMS agency–level association with variation in restraint or sedation use, there may be a role for improved standardization of prehospital restraint and sedation protocols for BHEs. Our results also suggest a need for increased EMS training on BHEs, and potentially consideration of expanded national prehospital education standards for BHEs that include de-escalation training.

### Limitations

Our study has several limitations. We used a clinical care dataset that relies on clinician-reported impressions. Our definition of BHE was more restrictive to avoid misclassifying patients with medical emergencies; thus, we likely excluded some patients with true BHEs. In addition, the available data did not include indications for restraint use, duration of restraint use, or attempts at verbal de-escalation before restraint or sedation. We also did not have access to measures of agitation severity. We cannot rule out that sedation was given for an indication other than agitation associated with a BHE; however, the results of our sensitivity analysis restricting the definition of sedation to medications administered intramuscularly were similar to the primary analyses, suggesting that potential encounters with sedation given for an indication other than agitation had a minimal association with findings. Additional patient factors, such as limited English proficiency, may have contributed to our findings, but information on such factors was not consistently available in our dataset. In this dataset, race and ethnicity were recorded based on EMS clinician impression, which may not always accurately capture a patient’s self-reported racial and ethnic identity. However, we believe EMS clinician impression is an accurate reflection of clinicians’ perceptions of the patient and potential bias. In secondary analyses, our finding that the non-Hispanic other group had lower odds of receiving chemical sedation or both restraint and sedation was unexpected. This was the smallest racial and ethnic subgroup, and our finding could reflect heterogeneity within this group—for example, different outcomes associated with Asian vs multiracial identity. Due to sample size limitations, we were unable to separately analyze the distinct racial groups within this category, but this remains an area for further study. Finally, the ESO research dataset is a sample of convenience, and the dataset has a geographic distribution weighted more toward the South US Census region and a higher proportion of urban responses compared with the nation. Despite these limitations, this nationwide study has important implications for prehospital care of patients with BHEs.

## Conclusions

In a large, nationwide dataset of EMS encounters, we identified racial and ethnic disparities in the prehospital use of physical restraint and/or chemical sedation for patients with BHEs. We found that non-Hispanic Black patients had significantly greater odds of being restrained and/or sedated across each of the outcomes evaluated. Clustering by EMS agency was associated with variation in restraint and/or sedation use. Future work should aim to better understand and address racial and ethnic disparities in the use of prehospital restraint and sedation, including potential investigation into EMS agency protocols for restraint and sedation and consideration of improvements to EMS protocols and trainings for management of patients with BHEs. Overall, the results of this study may guide initiatives to achieve more equitable care for patients with BHEs in the prehospital setting, which in turn has the potential to positively influence care for patients with BHEs in ED settings.

## Notes

Supplement 1. **eTable 1.** Inclusion Criteria for Behavioral Health Emergencies **eFigure.** Unadjusted Odds of Any Restraint/Sedation Use by Patient Race/Ethnicity **eTable 2.** Adjusted Odds of Restraint/Sedation in Sensitivity Analyses Restricting Definition of Chemical Sedation to Intramuscular Route

Supplement 2.
Data Sharing Statement
