# Supplementary material for: Racial and Ethnic Disparities in EMS Use of Restraints and Sedation for Patients With Behavioral Health Emergencies
Source: JAMA Netw Open. 2025 Mar 20;8(3):e251281. doi: 10.1001/jamanetworkopen.2025.1281 (PMC11926657; doi:10.1001/jamanetworkopen.2025.1281)
Supplement: Supplement 1. — eTable 1. Inclusion Criteria for Behavioral Health Emergencies eFigure. Unadjusted Odds of Any Restraint/Sedation Use by Patient Race/Ethnicity eTable 2. Adjusted Odds of Restraint/Sedation in Sensitivity Analyses Restricting Definition of Chemical Sedation to Intramuscular Route [file jamanetwopen-e251281-s001.pdf]

Supplementary Online Content

Bongiorno DM, Peters GA, Samuels-Kalow ME, et al. Racial and ethnic disparities in EMS use of restraints and sedation for patients with behavioral health emergencies. *JAMA Netw Open.* 2025;8(3):e251281. doi:10.1001/jamanetworkopen.2025.1281

eTable 1. Inclusion Criteria for Behavioral Health Emergencies
eFigure. Unadjusted Odds of Any Restraint/Sedation Use by Patient Race/Ethnicity
eTable 2. Adjusted Odds of Restraint/Sedation in Sensitivity Analyses Restricting Definition of Chemical Sedation to Intramuscular Route

This supplementary material has been provided by the authors to give readers additional information about their work.

© 2025 Bongiorno DM et al. *JAMA Network Open.*

eTable 1. Inclusion Criteria for Behavioral Health Emergencies
Study included encounters with a primary/secondary impression and/or protocol use and/or sign or symptom related to behavioral health emergency, as defined below.

Category	Inclusion criteria
Primary/secondary impression	anxiety reaction/emotional upset
	behavioral / psychiatric disorder
	behavioral/psychiatric episode
	mental disorder
	suicidal ideation
	suicide attempt
	alcohol dependence with withdrawal
	alcohol use
	cocaine related disorders
	other stimulant related disorders
	overdose - acetaminophen
	overdose - alcohol
	overdose - amphetamine
	overdose - benzodiazepine
	overdose - acetaminophen
	overdose - alcohol
	overdose - amphetamine
	overdose - benzodiazepine
	overdose - cannabis
	overdose - cocaine
	overdose - hallucinogens
	overdose - heroin
	overdose - methadone
	overdose - opium
	overdose - other opioids
	overdose - synthetic marijuana
	overdose - synthetic narcotics
	overdose - unspecified
	poisoning / drug ingestion
	sedative, hypnotic, or anxiolytic related disorders
	substance abuse
	substance/drug abuse
Protocol use	behavioral
	overdose / toxic ingestion
	opioid poisoning/overdose
Sign/symptom	auditory hallucinations
	bipolar disorder
	catatonic schizophrenia
	catatonic, refuses to respond
	central nervous system (stimulants) - adverse effect
	combative or violent behavior
	delusional disorders
	demoralization and apathy
	depression
	hallucinations
	homicidal ideations

© 2025 Bongiorno DM et al. *JAMA Network Open.*

	intoxication
	irritability and anger
	mental disorder, not otherwise specified
	opiates - adverse effect
	overactivity
	poisoning by unspecified antiepileptic and sedative-hypnotic drugs, undetermined
	poisoning by unspecified narcotics
	poisoning by unspecified psychotropic drug, undetermined
	psychotropic drugs - adverse effect
	restlessness and agitation
	strange and inexplicable behavior
	suicidal ideations
	toxic effect of unspecified substance, undetermined
	visual hallucinations
	withdrawal - alcohol
	withdrawal - psychoactive substance

© 2025 Bongiorno DM et al. *JAMA Network Open.*

eFigure. Unadjusted Odds of Any Restraint/Sedation Use by Patient Race/Ethnicity

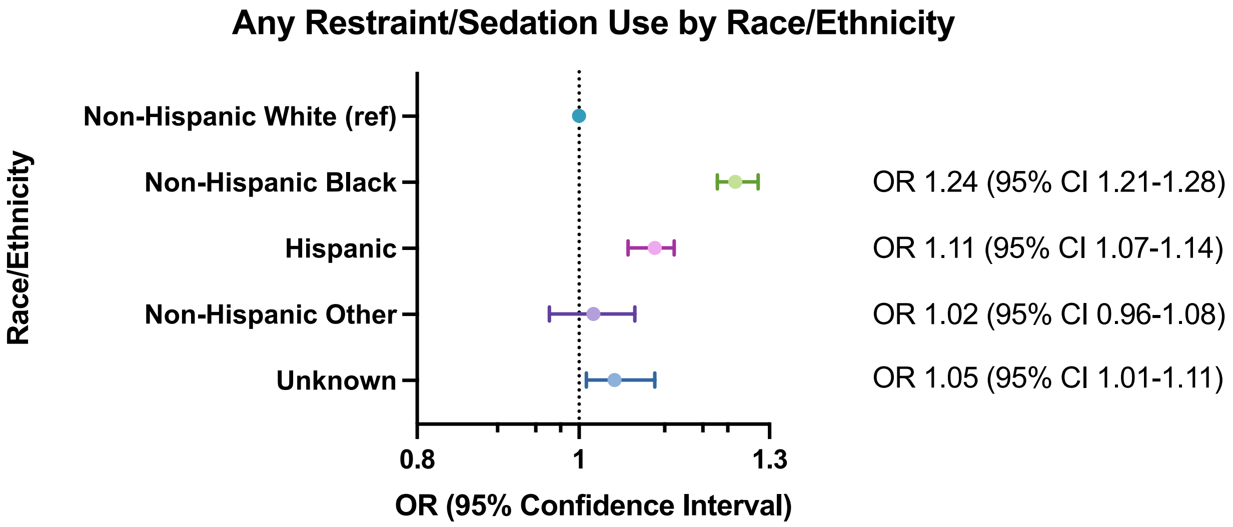

Models account for clustering by EMS agency.

© 2025 Bongiorno DM et al. *JAMA Network Open.*

eTable 2. Adjusted Odds of Restraint/Sedation in Sensitivity Analyses Restricting Definition of Chemical Sedation to Intramuscular Route

	Hispanic	Non-Hispanic Black	Non-Hispanic White	Non-Hispanic Other	Unknown
Any restraint and/or sedation	1.01 (95% CI 0.98-1.05)	1.24 (95% CI 1.20-1.27)	aOR[a] 1.0 (ref)	0.97 (95% CI 0.91-1.03)	1.04 (95% CI 0.99-1.09)
Any chemical sedation	0.90 (95% CI 0.85-0.96)	1.30 (95% CI 1.25-1.36)	1.0 (ref)	0.81 (95% CI 0.70-0.92)	1.06 (95% CI 0.98-1.16)
Both restraint and sedation	0.92 (95% CI 0.84-0.99)	1.34 (95% CI 1.25-1.43)	1.0 (ref)	0.76 (95% CI 0.63-0.93)	1.04 (95% CI 0.91-1.18)

[a]Adjusted odds ratio (aOR) are displayed throughout table. Models accounted for clustering by EMS agency and adjusted for age, gender, urbanicity, and community diversity (SVI minority status sub-theme).

© 2025 Bongiorno DM et al. *JAMA Network Open.*
