# Supplementary material for: Racial and Ethnic Disparities in EMS Use of Restraints and Sedation for Patients With Behavioral Health Emergencies
Source: JAMA Netw Open. 2025 Mar 20;8(3):e251281. doi: 10.1001/jamanetworkopen.2025.1281 (PMC11926657; doi:10.1001/jamanetworkopen.2025.1281)
Supplement: Supplement 2. — Data Sharing Statement [file jamanetwopen-e251281-s002.pdf]

Data Sharing Statement

Bongiorno. Racial and Ethnic Disparities in EMS Use of Restraints and Sedation for Patients With Behavioral Health Emergencies. *JAMA Netw Open*. Published March 20, 2025. doi:10.1001/jamanetworkopen.2025.1281

Data
Data available: No

Additional Information
Explanation for why data not available: The data utilized in this study were obtained from the ESO Data Collaborative, a national repository of prehospital and emergency care data. Access to this dataset is governed by strict data use agreements to ensure the security of the information and prevent gross misinterpretation of the data elements. Given these constraints, the dataset will not be made publicly available. However, researchers with a valid interest in the data may request access through the ESO Data Collaborative. Access will be granted following the submission and approval of a formal research proposal. Further details on the application process can be obtained by contacting ESO at Research@eso.com
